# CLRM-07. INCREASING EFFICIENCY IN EARLY PHASE MULTICENTER IMAGING BIOMARKER TRIALS: EMERGING STRATEGIES FROM THE GABLE (GLIOBLASTOMA ACCELERATED BIOMARKER LEARNING ENVIRONMENT) TRIAL

**DOI:** 10.1093/noajnl/vdab112.006

**Published:** 2021-09-21

**Authors:** Daniel Barboriak, Jon Steingrimsson, Constantine Gatsonis, David Schiff, Lawrence Kleinberg

**Affiliations:** 1 Duke University Medical Center, Department of Radiology, Durham, NC, USA; 2 Brown University, Department of Biostatistics, Providence, RI, USA; 3 University of Virginia Neuro-Oncology Center, Charlottesville, VA, USA; 4 Johns Hopkins University and The Sidney Kimmel Comprehensive Cancer Center, Baltimore, MD, USA

## Abstract

Validated biomarkers that more accurately predict prognosis and/or measure disease burden in patients with high-grade gliomas would help triage which treatment strategies are most promising for evaluation in Phase III multicenter trials. Multicenter trials to evaluate imaging biomarkers in this group face particular challenges; these trials have historically been slow to accrue and have not recently succeeded in validating new imaging biomarkers useful in treatment development. Due to variability in image acquisition protocols, scanner hardware, image analysis, and interpretive schemes, promising results obtained in single centers are poor predictors of success in the multicenter setting. Multicenter preliminary data to support further evaluation of imaging biomarkers is rarely available. The need for more efficient trial designs that bring multicenter data earlier into the process of biomarker development has become increasingly clear. In this presentation, the planning process within ECOG-ACRIN’s Brain Tumor Working Group for a platform multicenter trial called GABLE (Glioblastoma Accelerated Biomarker Learning Environment trial) designed to evaluate biomarkers for distinguishing pseudoprogression from true progression in patients with newly diagnosed GBM is described. In our planning process, it was determined that efficiencies can be gained from evaluating multiple biomarker types in parallel rather than serially; in the context of the proposed trial, not only conventional imaging biomarkers but plasma biomarkers and radiomic biomarkers can be evaluated simultaneously. Patient tolerance limits the feasibility of evaluating multiple non-standard-of-care imaging biomarkers in parallel. For this group of biomarkers, a “fast-switching” serial evaluation strategy using multiple interim analyses was developed to triage out biomarkers unlikely to succeed in identifying patient groups with clinically significant differences in median survival. For biomarker triage, an endpoint of event-free survival (events of either death or NANO progression) was proposed. Simulations were used to evaluate alpha and beta error using this evaluation strategy.

